# Clinical characteristics of oral chronic graft-versus-host disease according to the 2014 National Institutes of Health (USA) consensus criteria

**DOI:** 10.4317/medoral.25629

**Published:** 2022-09-29

**Authors:** Nawaporn Pengpis, Titipong Prueksrisakul, Chantiya Chanswangphuwana

**Affiliations:** 1 Postgraduate student. Oral Medicine Department, Faculty of Dentistry, Chulalongkorn University, Bangkok, Thailand; 2 Assistant Professor. Oral Medicine Department, Faculty of Dentistry, Chulalongkorn University, Bangkok, Thailand; 3 Division of Hematology, Department of Medicine, Faculty of Medicine, Chulalongkorn University and King Chulalongkorn Memorial Hospital, Bangkok, Thailand; 4 Research Unit in Translational Hematology, Chulalongkorn University, Bangkok, Thailand

## Abstract

**Background:**

Chronic graft-versus-host disease (cGVHD) is a serious and common complication of allogeneic hematopoietic cell transplantation (alloHCT). The oral cavity is the second most common site affected by cGVHD. In 2014, the 2005 National Institutes of Health (NIH) consensus criteria were revised to address areas of controversy. The aim of this study was to evaluate the clinical characteristics of oral cGVHD using the 2014 NIH consensus criteria.

**Material and Methods:**

The baseline data of oral manifestation of patients, who were diagnosed with oral cGVHD, in the first dental visit were analyzed (n=22). The oral mucosal disease was evaluated by NIH modified Oral Mucosa Rating Scale (OMRS) and Thongprasom sign score. The salivary gland disease and sclerotic disease were determined by the presence of signs and symptoms. The functional impact was assessed by the organ-specific severity score.

**Results:**

The median time from transplant to oral cGVHD diagnosis was 11.9 months. White striae with an erosive area was found in 72.7% of the patients. The mean ± SD of NIH modified OMRS was 6.1 ± 3.0. The most common and severely affected site of lichen planus-like features was buccal mucosa. Xerostomia, superficial mucocele and limited mouth opening were found in 18.2%, 9.1%, and 9.1%, respectively, of the patients. Almost all patients (90.9%) had partial limitation of oral intake. There were no significant differences in NIH modified OMRS or organ-specific severity score among the patient characteristic groups. Moreover, there was no association between the oral manifestations of cGVHD and the patient characteristics.

**Conclusions:**

The most common oral manifestation of cGVHD was white striae with an erosive area of oral mucosal disease, followed by xerostomia, superficial mucocele, and limited mouth opening. The 2014 NIH consensus criteria for diagnostic and severity assessment are informative and feasible in real-world practice.

** Key words:**Oral chronic graft-versus-host disease, 2014 National Institutes of Health consensus criteria, oral mucosal disease, hematopoietic cell transplantation.

## Introduction

Chronic graft-versus-host disease (cGVHD) is a serious and common complication of allogeneic hematopoietic cell transplantation (alloHCT), occurring in 30–70% of patients (1,2). Due to the increasing number of patients being treated with alloHCT for various hematologic diseases, and malignancies, cGVHD has become a major cause of late morbidity and mortality after transplantation (3). cGVHD is a clinical syndrome with variable features of signs and symptoms that resemble autoimmune and other immunological disorders. The manifestations of cGVHD may be restricted to a single organ or can involve multiple organs, approximately half of the affected patients have three or more involved sites, which impacts their quality of life (1,4). The oral cavity is the second most common site affected by cGVHD and has been reported in more than 70% of patients, presenting as oral mucosal lesions, salivary gland dysfunction, and restricted mouth opening (5,6).

In 2014, the 2005 National Institutes of Health (NIH) consensus criteria (7) were revised to address areas of controversy. The diagnostic criteria for the involvement of mouth, eyes, genitalia, and lungs were revised. The important changes in the recommendations in the oral section were the removal of hyperkeratotic plaques from the diagnostic features, the incorporation of asymptomatic lichen planus-like features in the organ-specific severity score, and the removal of a mucocele from the NIH modified Oral Mucosal Rating Scale (OMRS) assessment (1,8). Previous studies concerning the characteristics of oral cGVHD typically used the 2005 NIH consensus criteria (3,9,10). Therefore, the aim of this study was to evaluate the clinical characteristics of oral cGVHD using the revised 2014 NIH consensus criteria.

## Material and Methods

AlloHCT patients with oral cGVHD referred from the Hematology Division at the King Chulalongkorn Memorial Hospital, Thailand for evaluation and management of their oral cGVHD at the Oral Medicine Clinic, Faculty of Dentistry, Chulalongkorn University were enrolled in this retrospective study. The diagnosis of oral cGVHD was made by an oral medicine specialist by the presence of lichen planus-like features as 2014 NIH diagnostic criteria (1). Medical data was collected and evaluated from 2010-2021. The demographic data, patient characteristics and the oral manifestations of cGVHD were analyzed according to 3 components comprising oral mucosal disease, salivary gland disease, and sclerotic disease. The analyses were performed from the baseline data at the first dental visit. This study was approved by The Human Research Ethics Committee of the Faculty of Dentistry, Chulalongkorn University with study code HREC-DCU 2021-015, and the Institutional Review Board of Faculty of Medicine, Chulalongkorn University with COA No. 715/2021.

**Figure 1:**
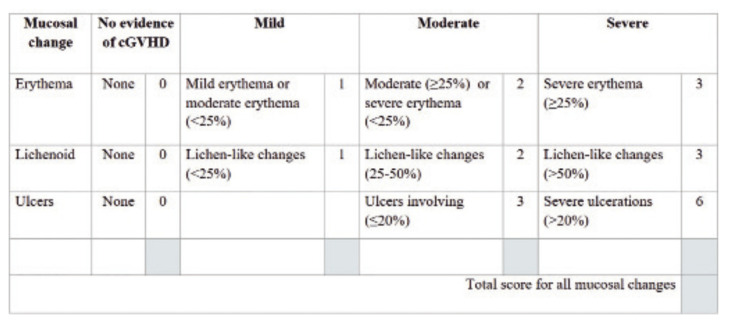
NIH modified Oral Mucosa Rating Scale (OMRS).

**Figure 2:**
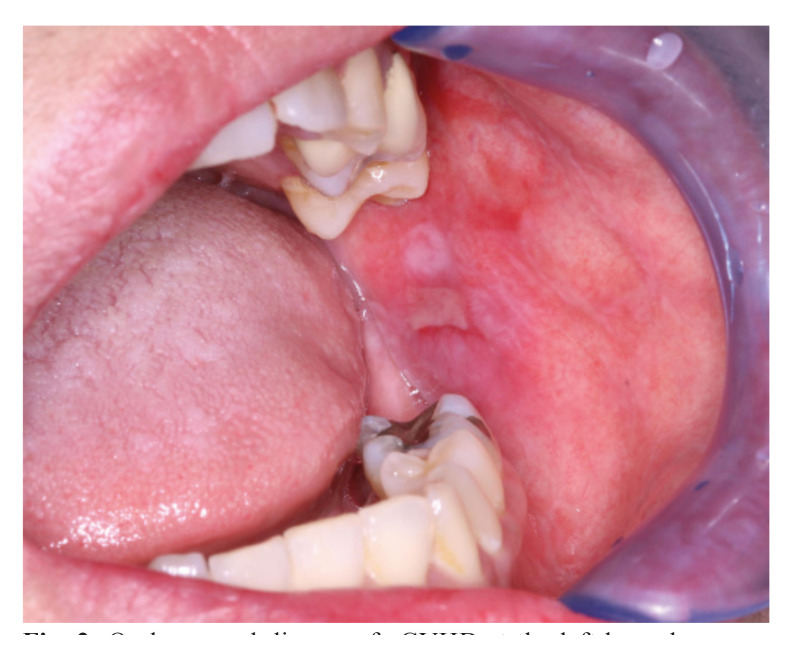
Oral mucosal disease of cGVHD at the left buccal mucosa with reticular white striae, erythematous, and ulcerative area.

The activity of the oral mucosal disease was determined by the NIH modified OMRS, which has an oral mucosal score ranging from 0–12. There are three manifestations assessed with this score a) mucosal erythema, b) lichenoid, and c) ulcerations (8) (Fig. 1). The Thongprasom sign score, the clinical score for the evaluation of the severity of oral lichen planus, was used to determine the severity and extension of the lichen planus-like changes in oral mucosal disease; erythema, white striae, and ulceration (Fig. 2).

The Thongprasom sign score is classified as score 0, no lesion; score 1, white striae only; score 2, white striae with an atrophic area less than 1 cm2; score 3, white striae with an atrophic area equal to or more than 1 cm2; score 4, white striae with an erosive area less than 1 cm2; score 5, white striae with an erosive area equal to or more than 1 cm2 (11).

**Figure 3:**
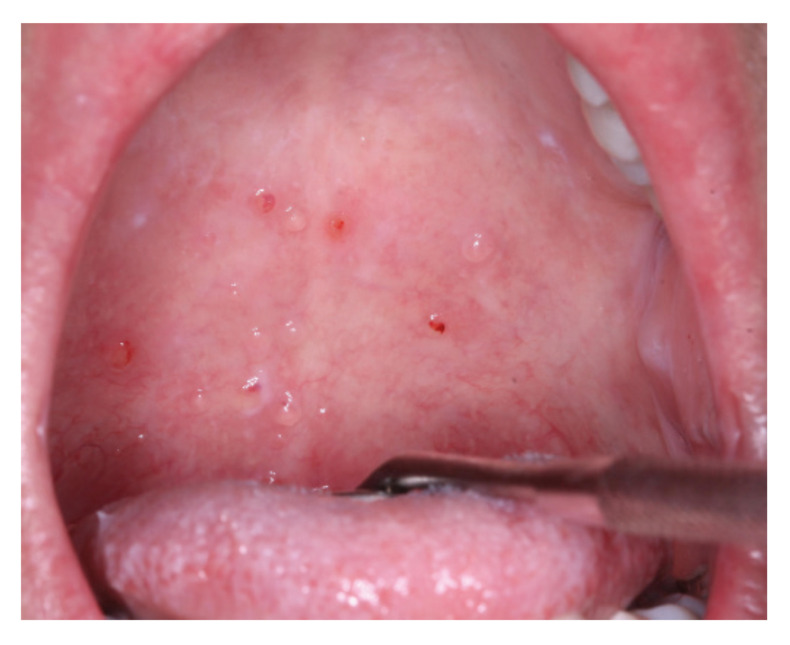
The presence of superficial mucoceles at the soft palate of oral cGVHD patient.

Salivary gland disease was evaluated by the subjective symptoms of the patient-reported xerostomia, and the presence of a superficial mucocele (Fig. 3). The history of the patient-reported symptoms about their restricted mouth opening and the signs of limited mouth opening due to sclerosis was evaluated in the sclerotic disease of oral cGVHD. The organ-specific severity scoring system was used to assess the severity and functional impairment of oral cGVHD, which has a 4–point scale (0–3), defined as score 0, no symptoms; score 1, mild symptoms with disease signs but not limiting oral intake significantly; score 2, moderate symptoms with disease signs with partial limitation of oral intake; score 3, severe symptoms with major limitations of oral intake (1).

The statistical analyses were performed using SPSS version 28 at the 0.05 significance level. Descriptive statistics were used to evaluate the demographic data, patient characteristics, and the oral manifestation of the cGVHD patients. The differences in the organ-specific severity score according to the patient characteristics were analyzed using the Mann-Whitney U test and the Kruskal-Wallis H test. The differences in the NIH modified OMRS according to the patient characteristics were investigated using the independent t-test and one-way ANOVA. The association of oral manifestations of cGVHD and patient characteristics was determined using Fisher's exact test or Fisher-Freeman-Halton exact test when appropriate.

## Results

The data of the 22 alloHCT patients with oral cGVHD were analyzed. All patients had undergone peripheral blood stem cell transplantation. Approximately 96% of patients were transplanted from HLA-matched donors. Almost all the patients were being treated with systemic immunosuppressive medication including prednisolone, cyclosporin, or tacrolimus. The patients’ main chief complaint was a burning sensation when eating hot or spicy food. The mean age at enrollment was 38.8 years old and 68.2% of patients were male. The median time from transplant to cGVHD diagnosis was 5.7 months. The median time from transplant to oral cGVHD diagnosis was 11.9 months. The median number of organ involvement was 2. The patients’ demographic data and characteristics are summarized in Table 1.

The presence of oral manifestations of cGVHD and organ-specific severity scores are presented in Table 2. The mean ± SD of NIH modified OMRS was 6.1 ± 3.0. The median highest Thongprasom sign score was 4 (IQR, 2.75–5.00). White reticular lesion with an erosive area was found in 72.7%. Xerostomia was reported in 18.2% of the patients. Superficial mucoceles were found in 9.1%. The restricted mouth opening was reported in 9.1% of the patients. The median organ-specific severity score was 2 (IQR, 2–2).

**Figure 4:**
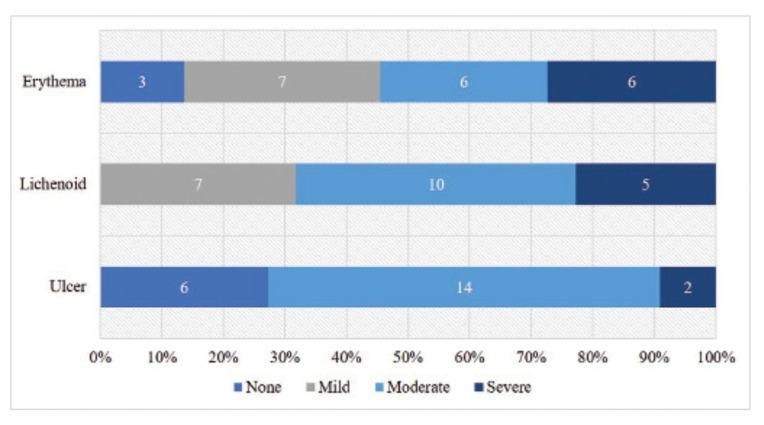
Frequency and disease activity in oral cGVHD patients as assessed by the NIH modified OMRS (*n*=22).

Table 3 demonstrates the severity and extension of the oral mucosal disease of cGVHD that were evaluated by the Thongprasom sign score. The most common site where the oral mucosal disease of cGVHD was involved in all patients was the buccal mucosa. The most severe site that presented with white striae with an erosive or ulcerative area equal to or more than 1 cm2 was also the buccal mucosa (27.3%). The lateral border of the tongue was the most common area that presented as only white striae were found in 59.1%. However, the soft palate was the least affected area that presented with no lesion in 86.4% of the patients. All of the patients presented with lichen planus-like changes, of which 31.8% were mild, 45.5% were moderate, and 22.7% were severe. Approximately 73% of the patients had ulcerations (Fig. 4).

**Table 1:**
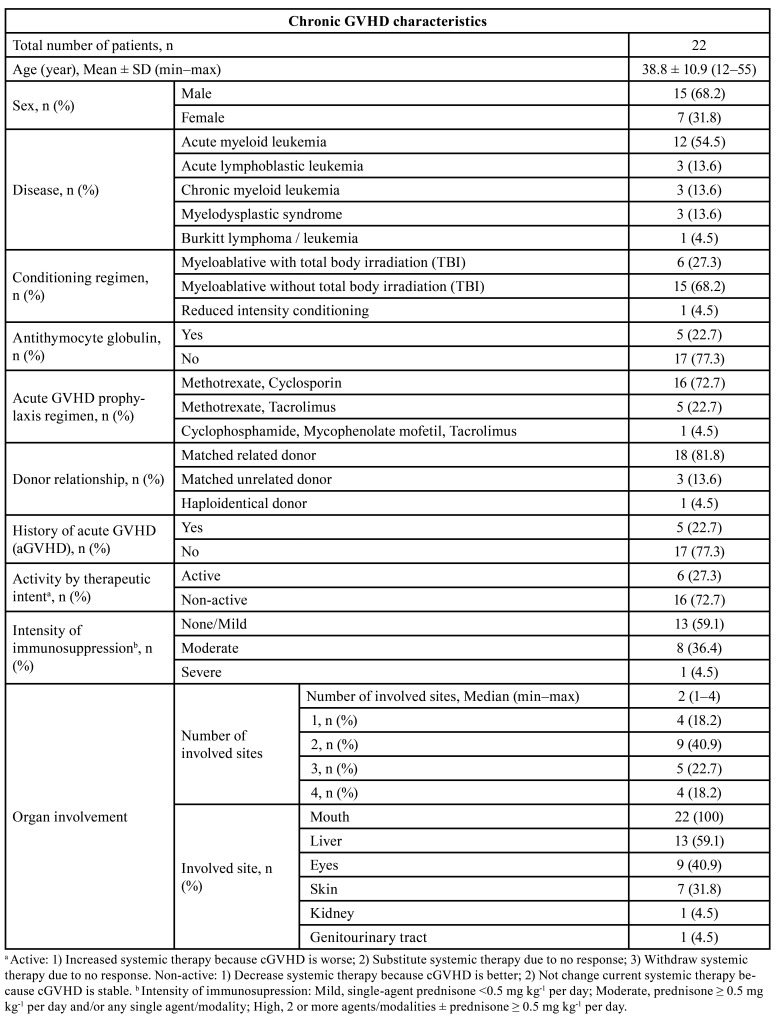
Demographic data and patient characteristics.

**Table 2:**
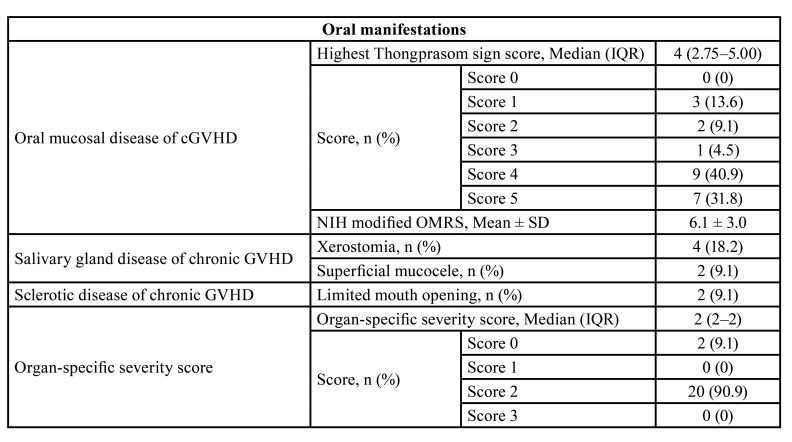
Oral manifestations of cGVHD and organ-specific severity score.

**Table 3:**
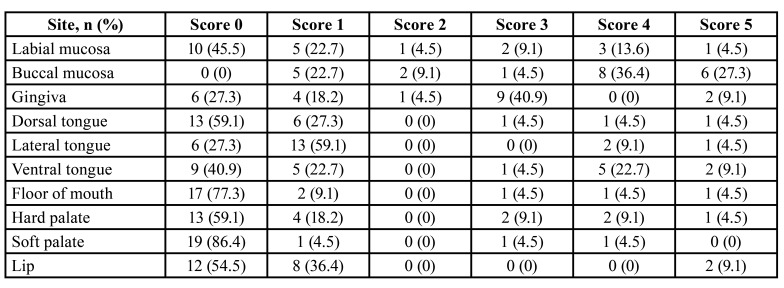
he severity and extension of the oral mucosal disease of cGVHD by Thongprasom sign score.

The NIH modified OMRS and organ-specific severity score according to patient characteristics were compared. The analyses demonstrated there were no significant differences in NIH modified OMRS or organ-specific severity score among the patient characteristics groups. Fisher's exact test and the Fisher-Freeman-Halton exact test indicated no association between the oral manifestations of cGVHD and the patient characteristics.

## Discussion

Oral cGVHD has a meaningful impact on the quality of life of the patient, especially concerning the limited food intake. In this study, we found that 90.9% of the patients had moderate symptoms with disease signs with a partial limitation of oral intake because of burning sensation in their mouth. These symptoms would arise due to having an oral ulceration, which conforms to our findings. Furthermore, the presence of ulcerations involved the oral mucosa, which was assessed using the NIH modified OMRS, and the highest Thongprasom sign score was score 4 or 5 in 72.7% of the patients. We also found that the site most affected by oral mucosal disease of cGVHD was the buccal mucosa, which is similar to that of a prior study by Scaraficci AC *et al*. (12). Moreover, we found the most severely affected site that presented with ulcerative lesions was also the buccal mucosa, followed by the ventral tongue. Therefore, our findings can be helpful information for health care providers and alloHCT patients to observe and evaluate the typically affected areas of oral mucosal disease of cGVHD.

In this study, we found only 18.2%, 9.1%, and 9.1% of the patients with xerostomia, a superficial mucocele, and limited mouth opening, respectively. A previous study of the factors associated with salivary dysfunction in cGVHD found that none of the demographic and transplant parameters were predictive of salivary gland dysfunction (13). In contrast, Hull KM *et al*. found a significant inverse association between the time since transplantation and the degree of salivary hypofunction. Moreover, they found an association between the degree of salivary hypofunction and the history of aGVHD, of which the severity of salivary hypofunction was greater in the history of aGVHD group. Furthermore, they found no association was found between xerostomia and the time since transplantation, history of aGVHD, stem cell source, or type of hematopoietic cell transplantation (14). In our study, none of the four xerostomia patients had a history of aGVHD.

Busca A *et al*. reported that the median time from the onset of cGVHD to the appearance of oral manifestations was 68 days, (range 5–775 days) (15). In contrast, we found that the median number of months from transplant to oral cGVHD diagnosis was 11.9 months, (range 3.8–39.7 months, 114–1190 days). The reason for this difference is that in our study, some patients did not come to our clinic upon the emergence of their symptoms. Our findings suggest that the optimum time to initiate oral cGVHD screening is approximately 100 days after transplantation. Moreover, the recommendations for the early recognition of cGVHD in the 2020 NIH consensus criteria have also been suggested for assessing cGVHD manifestations at baseline before transplantation, approximately 100 days after transplantation, and follow-up every 1–3 months until the patient has discontinued the immunosuppressive treatment for at least 6 months to prevent evolution to more severe disease with irreversible damage (16).

The limitations of this study were that this study was conducted as a single-center retrospective study and had a small sample size (*n*=22), which would be a limitation for data analysis. Furthermore, our study was a retrospective study design that evaluate the baseline oral cGVHD by 2014 NIH consensus criteria. Additional studies performed in a larger group with a multicenter, prospective study design aim to assess the treatment response and long-term outcomes using these standard international criteria.

Our findings revealed that there were no differences in organ-specific severity score or NIH modified OMRS among the patient characteristic groups and we found no association between the oral manifestations of cGVHD and the patient characteristics. However, our study intensively reviewed and comprehensively analyzed of oral cGVHD data with various functional and clinical scores, which followed the revised 2014 NIH consensus criteria. Our findings represent the clinical characteristics of patients with oral cGVHD in multidimensions that will be useful primary data for use in future studies.

## Conclusions

The most common oral manifestation of cGVHD was white striae with an erosive area of oral mucosal disease, followed by xerostomia, superficial mucocele, and limited mouth opening. However, this study found no differences in the organ-specific severity score or the NIH modified OMRS among the patient characteristic groups. Furthermore, there was no association between the oral manifestations of cGVHD and patient characteristics. The 2014 NIH consensus criteria for diagnostic and severity assessment are informative and feasible in real-world practice.
